# Social context during moral decision-making impacts males more than females

**DOI:** 10.3389/fpsyg.2024.1397069

**Published:** 2024-05-21

**Authors:** June J. Pilcher, Phillip D. Smith

**Affiliations:** Department of Psychology, Clemson University, Clemson, SC, United States

**Keywords:** moral judgments, moral dilemmas, gender differences, social context, social judgment, morality, moral reasoning difference score

## Abstract

Moral judgments are often viewed as the outcome of affective and deliberative processes that could be impacted by social factors and individual characteristics. The purpose of this study was to examine the interaction between gender and social context on moral judgment. Participants included 315 undergraduate students (67.3% female). The participants completed the Moral Decision-Making Task while seated at row tables facing the front of the room or round tables facing other participants. The results indicated that males responded in a more utilitarian manner (harm one to save five) than females for moral impersonal (MI) and moral personal (MP) dilemmas regardless of seating arrangements. When seated at round tables, all participants were more likely to respond deontologically (cause no harm) to the moral impersonal dilemmas. In addition, we calculated a moral reasoning difference score for each participant as the difference between the MI and MP scores to represent additional reactivity due to the idea of taking direct action. The moral reasoning difference score was consistent for females but indicated a more deontological response from males at round tables and a more utilitarian response from males at row tables. These results suggest that males are more utilitarian than females and are more likely to be influenced by social context when responding to moral dilemmas. More broadly, the current results indicate that moral judgments are affected by social context particularly in males in ways that have not been incorporated in many models of moral decision making.

## Introduction

Considerable research has investigated moral judgment by examining participant responses to moral scenarios such as the classic trolley problem (Foot, 1967; Waldmann et al., 2012). Traditionally, there have been two versions of the trolley problem, one that requires the participant to decide whether to move a switch to divert a trolley such that the trolley kills one person but saves five workers on the original track (moral impersonal scenarios). The other version requires the participant to decide whether to push a person off a footbridge into the path of the trolley thus saving five workers on the track (moral personal scenarios). The two versions are specifically designed to be decontextualized and to differ in terms of the amount of direct action required of the participant when choosing to sacrifice one person to save others such that moving a switch is a more detached action than pushing the person from a bridge. In either scenario, choosing to sacrifice one person for the greater good of saving five people is referred to as a utilitarian response while choosing not to harm the bystander despite the possibility of saving five people is referred to as a deontological response.

Utilitarian and deontological responses to moral scenarios are often considered to be driven by a dual-processing cognitive system (Greene et al., 2001, 2004). Dual-process models suggest that human decision-making results from an interaction between deliberative and affective cognitive responses. Based on the dual-process model, utilitarian choices are viewed as a deliberate or logical assessment of potential outcomes which can result in the decision to harm one person for the greater good. In contrast, deontological choices are viewed as the result of a negative affective response to the potential of harming someone independent of the number of people who might benefit. The evoked affective reactivity that is part of a deontological response often leads people to desire inaction when responding to moral scenarios, while utilitarianism often leads people to desire action (Koenigs et al., 2007).

Although there is increasing evidence that moral judgments such as those made for the classic trolley problem depend on multiple factors, moral decision making is often discussed primarily as a dual process reflecting a trade-off between affective responding and purposeful deliberation. Some research suggests that the focus on dual processing when interpreting moral judgment is not incorrect but may not account for possible mitigating factors (Byrd and Conway, 2019). A number of researchers have postulated that motivational, situational, and contextual factors (Björklund, 2003; Bartels et al., 2015; Schein, 2020; McHugh et al., 2022) as well as numerous individual differences (Graham et al., 2011) could affect moral judgment. Furthermore, research suggests that people are not solely utilitarian or deontological. Instead, most people seem to take different aspects of the decision-making scenario into account when they choose an action (Bartels et al., 2015).

Research indicates that in addition to affective and deliberative processes, people are influenced by social context when making moral decisions. The moral judgment itself is fundamentally social in nature in that one must consider other people as part of the decision process (Haidt, 2007). Moreover, humans are aware of and concerned about how others perceive them when in a social setting. People want their public persona to be perceived as matching the values and preferences of important others (Leary and Kowalski, 1990) and will present themselves in different ways to conform to social expectations (Leary, 2019). Research indicates that social situations also influence responses to moral dilemmas. For example, social pressure has been shown to influence dilemma judgments (Lucas and Livingston, 2014; Bostyn and Roets, 2016). One study found that participants used dilemma judgments to appear trustworthy to a partner (Everett et al., 2016) in that the participants may have chosen more deontological responses on some moral dilemmas to avoid appearing amoral when resolving dilemmas in front of another person. Another study found an increase in deontological judgments in participants who were told that they were being monitored and their responses recorded while completing the moral dilemmas in comparison to non-monitored participants (Lee et al., 2018). Furthermore, Rom and Conway found that people anticipate how others will view them based on their dilemma judgments and can shift their public dilemma judgments to present themselves in a more acceptable manner depending on the social situation (Rom and Conway, 2018).

Some evidence suggests that gender could also impact moral decision making. It is a common stereotype that men and women differ in their moral orientations with men often being viewed as more rational and morally superior while women are viewed as more intuitive and empathetic (Brabeck, 1983). Although some early studies concluded that there were differences in how males and females make moral decisions, the evidence was mixed and may have been the result of inconsistent methods used in gathering data (Brabeck, 1983; Walker, 1984; Ford and Lowery, 1986). More recent literature is again evaluating the potential impact of gender in moral decision making. One study found that mindset influences moral judgments in males but not females (Hügelschäfer and Achtziger, 2014). Other studies have found gender differences on dilemma judgements where men choose more utilitarian responses to personal moral dilemmas than women suggesting that women were more likely to reject harming others (Fumagalli et al., 2010; Arutyunova et al., 2016; Capraro and Sippel, 2017). A meta-analytic review also found gender differences where women exhibited stronger deontological responses than men and that these decisions were due to affective responses to potential harm more than a deliberate evaluation of potential outcomes (Friesdorf et al., 2015). However, little research has examined the potential impact of social context on how males and females react when making moral decisions.

The purpose of the current study was to examine the potential interaction between gender and social context on moral decision making. Social context in this study was based on the seating arrangement where participants were seated either in a classroom with rows of tables facing the front of the room or in a classroom with round tables facing each other. To examine moral decision making, we averaged the responses for the moral impersonal scenarios to create a moral impersonal score and the responses on the moral personal scenarios to create a moral personal score. We also calculated a moral reasoning difference score that was the difference between the moral impersonal and moral personal scores as an indicator of potential reactivity to the moral personal scenarios beyond the reactivity to the moral impersonal scenarios. Based on the literature, we tested the following hypotheses. [H1] We hypothesize that males will respond with more utilitarian actions than females for moral personal dilemmas. [H2] Although there is less literature examining the potential effect of seating arrangements, we hypothesize that the face-to-face seating arrangement will result in increased deontological responding on moral dilemmas given that people often seek approval from others and may not want to appear to be amoral. We are unable to derive hypotheses on potential interactions between gender and the social context resulting from the seating arrangements due to a lack of literature examining this type of social context manipulation. Similarly, because previous studies have not reported a variable similar to the calculated moral reasoning difference score, we are unable to generate hypotheses for this variable.

## Methods

### Participants

Three hundred and fifteen undergraduate students (*M* = 18.44, *SD* = 0.98 years, 212 females) took part in the study. All participants completed a screening survey and were in good mental and physical health. The Institution Review Board approved the study and each participant provided consent prior to data collection. All participants were enrolled in an introductory psychology course and received research course credit for completing the study.

### Measures

Moral reasoning was assessed using the Moral Decision-Making Task (MDMT). The MDMT uses hypothetical scenarios such as the trolley problem described previously and requires the participants to make decisions about the appropriateness of proposed actions based on the scenarios that will directly or indirectly lead to the injury or death of one person to save multiple people from injury or death. The current study modelled previous studies (Koenigs et al., 2007; Sylvia et al., 2013) and used 20 moral dilemmas: 5 moral impersonal (MI) dilemmas and 15 moral personal (MP) dilemmas. The MP dilemmas require the person to imagine taking direct action that touches the person and evoke significantly more emotion than the MI dilemmas (Koenigs et al., 2007).

### Procedures

Participants signed up for one of 10 possible testing sessions. Each testing session took place between 1030 and 1730. Two types of standard university classrooms were used for data collection. One classroom was set up in a standard row configuration where all students faced the front of the classroom (face-forward condition). The second room was set up with circular tables with each table seating eight persons requiring students to be seated across from each other (face-to-face condition). One hundred and thirty-nine participants completed the study in the face-forward condition (*M* = 18.5, *SD* = 1.1 years, 92 females). One hundred and seventy-six participants completed the study in the face-to-face condition (*M* = 18.45, *SD* = 0.86 years, 120 females). After arrival in the testing room, participants used their personal laptop to access an electronic form with information about the study, provide consent, complete the demographics form, and complete the MDMT. The 20 scenarios used in the MDMT were presented in counterbalanced order. Data collection took approximately 10 min. Data collection was completed by one of two trained researchers using a specific protocol that designated each step of the experimental process. The protocol included the specific order of tasks and the verbiage to use when addressing the participants.

### Data analysis

The MDMT was scored in accordance with previous research (Moore et al., 2008), resulting in two MDMT-based metrics. For the MI scenarios, the mean proportion of responses for each participant that indicated approval of the proposed utilitarian action (e.g., moving a switch to harm one person but save five persons) provided the MI score. For the MP scenarios, the mean proportion of responses for each participant that indicated approval of the proposed utilitarian action (e.g., harming one person directly to save five persons) provided the MP score. In addition, we calculated a moral reasoning difference (MRD) score for each participant as the difference between the MI and MP scores. We suggest that the MRD score represents the extent of reactivity elicited from the MP scenarios beyond the reactivity elicited from the MI scenarios.

The data were analyzed using IBM SPSS 22 (SPSS Inc., Chicago, IL). A 2 (gender) x 2 (condition: face-forward or face-to-face) ANOVA was conducted for each metric derived from the MDMP: the MI, MP, and MRD scores.

## Results

The means, standard deviations, and ANOVA results for the MI score are shown in Table 1. There was a significant main effect for both gender and condition for the MI score. Males had significantly higher MI scores than females indicating a greater utilitarian response. In addition, all participants in the face-to-face condition had significantly lower MI scores indicating a greater deontological response than the participants in the face-forward condition. Although it approached significance, there was no significant interaction for the MI scores.

**Table 1:** MI score.

Gender	Condition	Mean	SD	df	*F*	*p*	η_p_^2^
Male		0.804	0.242	1, 311	4.041	0.045*	0.013
Female		0.747	0.239				
	F-to-F	0.732	0.237	1, 311	8.795	0.003*	0.028
	F-forward	0.800	0.241				
Male	F-to-F	0.736	0.264	1, 311	3.278	0.071	0.010
	F-forward	0.872	0.189				
Female	F-to-F	0.730	0.224				
	F-forward	0.763	0.257				

*Significant *p*-values. F-to-F, face-to-face condition. F-forward, face-forward condition.

The means, standard deviations, and ANOVA results for the MP score are shown in Table 2. There was a significant main effect for gender with males having significantly higher MP scores indicating a greater utilitarian response than females. There was no significant effect for condition or interaction for the MP scores.

**Table 2:** MP score.

Gender	Condition	Mean	SD	df	*F*	*p*	η_p_^2^
Male		0.442	0.201	1, 311	5.360	0.021*	0.017
Female		0.389	0.184				
	F-to-F	0.405	0.015	1, 311	0.858	0.355	0.003
	F-forward	0.426	0.017				
Male	F-to-F	0.433	0.222	1, 311	0.024	0.878	0.000
	F-forward	0.451	0.174				
Female	F-to-F	0.377	0.178				
	F-forward	0.401	0.192				

*Significant *p*-values. F-to-F, face-to-face condition. F-forward, face-forward condition.

The means, standard deviations, and ANOVA results for the MRD score are shown in Table 3. There was no main effect of gender for the MRD scores. There was a main effect for condition with all participants seated face-to-face responding with lower MRD scores indicating a greater deontological response than participants seated facing forward. The interaction for MRD was also significant with males having lower MRD scores indicating a more deontological response than females in the face-to-face condition but higher scores indicating a more utilitarian response than females in the face-forward condition.

**Table 3:** MRD score.

Gender	Condition	Mean	SD	df	*F*	*p*	η_p_^2^
Male		0.362	0.217	1, 311	0.025	0.874	0.000
Female		0.357	0.235				
	F-to-F	0.337	0.227	1, 311	5.35	0.021*	0.017
	F-forward	0.382	0.230				
Male	F-to-F	0.302	0.211	1, 311	4.05	0.045*	0.013
	F-forward	0.421	0.209				
Female	F-to-F	0.353	0.233				
	F-forward	0.362	0.239				

*Significant *p*-values. F-to-F, face-to-face condition. F-forward, face-forward condition.

## Discussion

The current study examined the interaction between social context and gender on moral decision making. The results indicated that males provided more utilitarian responses than females on both the MI and MP scenarios. However, there was no gender effect on the MRD score. The current results also showed that the face-to-face condition resulted in a more deontological response from both females and males for the MI scenarios and the MRD score but not the MP scenarios. The only significant interaction effect was found on the MRD score where males were more deontological than females in their responses in the face-to-face condition but more utilitarian than females in their responses in the face-forward condition.

Our first hypothesis was supported in that males were more utilitarian than females when reacting to MP dilemmas in the current study thus supporting previous literature (Fumagalli et al., 2010; Arutyunova et al., 2016; Capraro and Sippel, 2017). The current results also indicate that males were more utilitarian than females when reacting to MI dilemmas. This contrasts with the previous finding that males did not respond in a more utilitarian way than females to MI dilemmas (Fumagalli et al., 2010). These different results could reflect differing social or moral norms as well as different age groups (Arutyunova et al., 2016). Additional research is needed that more fully addresses potential gender-related differences in moral decision making.

The current results also partially supported our second hypothesis. The finding that the participants in the face-to-face condition reacted in a more deontological manner for MI dilemmas supports previous literature suggesting that people may alter their moral decisions based on the context where the decisions are being made (Bartels et al., 2015; Rom and Conway, 2018; McHugh et al., 2022). The current study focused specifically on the context and not the broader aspects of social interactions. In this case, the participants were not actually interacting or imagining interacting, they were simply facing each other while working on their laptops. This suggests that seeing a nearby person’s face is enough of a stimulus to cause a more deontological response on the MI dilemmas.

It is interesting that the MP dilemmas did not result in a greater deontological response in the face-to-face condition. It is possible that the emotional-laden content of the MP dilemmas was more resistant to change (Lanteri et al., 2008) and was less impacted by any extra effect that could incur from facing another person across a table. The derived MRD score may help address this issue by accounting for affective reactivity to moral dilemmas as specified by the MI scores. The participants in the face-to-face condition responded in a more deontological manner as indicated by the MRD scores. This suggests that when we account for the reaction to a moral scenario requiring one to consider an unpalatable response, seating facing others creates a context that results in a larger deontological reaction to moral dilemmas than when seated facing the back of the heads of the other participants.

The differential response seen in the MRD scores between females and males is due to the males modifying their responses to the dilemmas based on the classroom setting. In the current study, females exhibited more consistent moral reasoning than males as indicated by the MRD scores across the two seating conditions. In contrast, the MRD scores in males indicated greater deontological-based responding than females when seated at round tables facing other participants but greater utilitarian-based responding when seated facing the front of the room. These results support previous research suggesting that moral judgment in females is more stable than in males (Humphries et al., 2000; Alhola and Polo-Kantola, 2007) as well as the assumption that female responses to moral dilemmas reflect internalized values whereas males more readily modify their responses based on external cues (Hoffman, 1975).

There are several possible explanations of these findings. Females may base their moral decisions on their personal standards or a more accurate internal recognition of affective reactivity than males resulting in a more stable moral decision that is not readily impacted by the social context. Alternatively, females may feel less willing to adjust their moral judgments in ways that may conflict with social norms and expectations of others perhaps due to socialization and social expectations (Bussey and Maughan, 1982; Eagly and Wood, 1999; Rudman and Glick, 1999). In contrast, male responses may result from an interaction between internal and external cues. For example, a higher basal testosterone level may contribute to a willingness to make utilitarian judgments in males since testosterone levels correspond with lower aversion to risk (Carney and Mason, 2010). Males also place more value on principles of justice and fairness (Fumagalli et al., 2010) which could lead to increased likelihood of utilitarian responses. However, males may be more likely to conform to social norms to not hurt others in situations where they perceive that they may be judged for such a reaction. In the current study, males may have reacted as if other participants could judge them even when the person is simply seated across a table working on their own tasks. To better differentiate between these types of possible explanations, further research is needed on the potential interaction between gender and social context in moral decision making. Furthermore, the derived MRD score reported in the current study provided a viable mechanism to better understand gender-related reactivity to MI and MP scenarios in different social contexts. Additional research is needed that reports and interprets the MRD score to provide comparative measures in other studies.

The current results support previous research concluding that different contexts and social settings can impact moral decision making (Bartels et al., 2015; Rom and Conway, 2018; McNamara et al., 2019). Moral dilemmas exist due to broad psychological processes that allow humans to consider potentially divergent responses (Cushman, 2013). The diversity of this psychological process provides many options for individuals facing moral decisions that may not be straightforward to delineate. In general, moral judgments can be considered a result of specific processes (e.g., affect and logic) as well as being adaptable to different contexts and settings (Rom and Conway, 2018). More specifically, the current results suggest that males may be more reactive to different social contexts when responding to moral dilemmas than females.

The current study has some limitations. The validity of decontextualized hypothetical scenarios as a proxy for measuring real-life moral decision making has been questioned (FeldmanHall et al., 2012). Although the moral dilemmas used in the current study are not designed to reflect actual moral decisions typically made in daily life, the decontextualized approach used here provides for reliable testing of factors that may impact moral judgments and are relevant for many types of research questions (Schein, 2020). Future studies can be designed to test whether the current results are supported using more realistic moral scenarios. Another limitation is that we did not include any type of subjective reports on whether participants felt that they were being observed in some way by other participants. One of the strengths of the current study is that the interaction when seated at the round tables was minimal to non-existent between the participants and yet just being seated in a circle facing others had an impact on moral judgment. Researchers designing future studies could consider adding self-report measures at the end of the study about the feeling of being observed while completing the tasks. A third limitation is that the current study did not randomly assign participants to the room conditions. Instead, participants were allowed to match their academic schedule with testing times that were preset by room availability. Although this is not true random assignment, the type of classroom setting was unlikely to be the main concern for the participants when signing up for the study. It seems more reasonable that the participants were most concerned about fitting the time of the study into their schedules. Future studies can be designed to randomly assign participants to the specific room type to better control for this potential issue. Finally, we did not attempt to control how the participants were seated in either classroom setting. It is possible, particularly in the face-to-face condition, that the sex of the participants seated nearby or across the table could impact responses to the moral dilemmas. Future studies are needed that manipulate the sex composition of the seating arrangements. For example, a study could use a face-to-face seating condition where participants were always facing a person of the opposite sex or even at a table with the other participants all being the opposite sex. Another possibility is to set up the testing sessions so that the entire room or individual tables consist of solely one sex to examine if single-sex seating arrangement leads to different moral judgments than mixed-gender seating arrangements. Despite these possible limitations the current study provides insight into the impact of social context in terms of seating arrangements and gender on moral decision making that is not yet well documented in the literature.

## Conclusion

This is the first study to our knowledge that examines how females and males react to moral dilemmas in different social contexts represented by two types of seating arrangements that are commonly used in educational and work settings. We also provide a way to document moral decision making by proposing the calculation of the MRD score as an indication of reactivity elicited by the MP scenarios above that elicited by the MI scenarios. The current results build on previous research suggesting that males are less likely than females to be influenced by evoked reactivity to moral dilemmas and that females exhibit more stable moral decision making than males. Furthermore, the results indicate that when seated facing others, both females and males respond in a more deontological manner to moral dilemmas. The current results also add that when seated facing others, males are more likely to make an affective-based decision whereas when seated facing the front of the room, males are more likely to make logic-based decisions. This set of results suggest that moral judgments rely on more than only affective reactions or rational decision making. Social contexts that may engage higher order decision processing may induce flexibility in moral judgments that has not yet been well integrated into the classic models of moral decision making. This suggests a level of complexity to moral decision making that requires additional investigation. The current results indicate that moral judgments are not based solely on a simple dichotomy of good versus bad or affect versus logic. To better understand how individuals make moral judgments, researchers should endeavor to better account for the variability and complexity intrinsic in moral decision making.

## Data availability statement

The raw data supporting the conclusions of this article will be made available by the authors, without undue reservation.

## Ethics statement

The studies involving humans were approved by Clemson University Institution Review Board. The studies were conducted in accordance with the local legislation and institutional requirements. The participants provided their written informed consent to participate in this study.

## Author contributions

JP: Writing – review & editing, Writing – original draft, Visualization, Supervision, Resources, Project administration, Methodology, Investigation, Funding acquisition, Formal analysis, Conceptualization. PS: Writing – review & editing, Writing – original draft, Methodology, Investigation, Formal analysis, Data curation, Conceptualization.

## References

[ref1] Alhola P. Polo-Kantola P. (2007). Sleep deprivation: impact on cognitive performance. Neuropsychiatr. Dis. Treat. 3, 553–567 doi: 10.2147/ndt.s12160203, PMID: 19300585 PMC2656292

[ref2] Arutyunova K. R. Alexandrov Y. I. Hauser M. D. (2016). Sociocultural influences on moral judgments: east–west, male–female, and young–old. Front. Psychol. 7:1334. doi: 10.3389/fpsyg.2016.01334 27656155 PMC5011137

[ref3] Bartels D. M. Bauman C. W. Cushman F. A. Pizarro D. A. McGraw A. P. (2015). “Moral judgment and decision making” in The Wiley Blackwell handbook of judgment and decision making. eds. Keren G. Wu G. (Chichester, UK: Wiley Online Library), 478–515.

[ref4] Björklund F. (2003). Differences in the justification of choices in moral dilemmas: effects of gender, time pressure and dilemma seriousness. Scand. J. Psychol. 44, 459–466. doi: 10.1046/j.1467-9450.2003.00367.x, PMID: 15030112

[ref5] Bostyn D. H. Roets A. (2016). The morality of action: the asymmetry between judgments of praise and blame in the action–omission effect. J. Exp. Soc. Psychol. 63, 19–25. doi: 10.1016/j.jesp.2015.11.005

[ref6] Brabeck M. (1983). Moral judgment: theory and research on differences between males and females. Dev. Rev. 3, 274–291. doi: 10.1016/0273-2297(83)90016-3

[ref7] Bussey K. Maughan B. (1982). Gender differences in moral reasoning. J. Pers. Soc. Psychol. 42, 701–706. doi: 10.1037/0022-3514.42.4.701

[ref8] Byrd N. Conway P. (2019). Not all who ponder count costs: arithmetic reflection predicts utilitarian tendencies, but logical reflection predicts both deontological and utilitarian tendencies. Cognition 192:103995. doi: 10.1016/j.cognition.2019.06.007, PMID: 31301587

[ref10] Capraro V. Sippel J. (2017). Gender differences in moral judgment and the evaluation of gender-specified moral agents. Cogn. Process. 18, 399–405. doi: 10.1007/s10339-017-0822-9, PMID: 28597324

[ref11] Carney D. R. Mason M. F. (2010). Decision making and testosterone: when the ends justify the means. J. Exp. Soc. Psychol. 46, 668–671. doi: 10.1016/j.jesp.2010.02.003

[ref12] Cushman F. (2013). Action, outcome, and value: a dual-system framework for morality. Personal. Soc. Psychol. Rev. 17, 273–292. doi: 10.1177/1088868313495594 23861355

[ref13] Eagly A. H. Wood W. (1999). The origins of sex differences in human behavior: evolved dispositions versus social roles. Am. Psychol. 54, 408–423. doi: 10.1037/0003-066X.54.6.408

[ref14] Everett J. A. Pizarro D. A. Crockett M. J. (2016). Inference of trustworthiness from intuitive moral judgments. J. Exp. Psychol. Gen. 145, 772–787. doi: 10.1037/xge0000165, PMID: 27054685

[ref15] FeldmanHall O. Mobbs D. Evans D. Hiscox L. Navrady L. Dalgleish T. (2012). What we say and what we do: the relationship between real and hypothetical moral choices. Cognition 123, 434–441. doi: 10.1016/j.cognition.2012.02.001, PMID: 22405924 PMC3355304

[ref16] Foot P. (1967). The problem of abortion and the doctrine of the double effect. Oxford Review 5, 1–7,

[ref17] Ford M. R. Lowery C. R. (1986). Gender differences in moral reasoning: a comparison of the use of justice and care orientations. J. Pers. Soc. Psychol. 50, 777–783. doi: 10.1037/0022-3514.50.4.777

[ref18] Friesdorf R. Conway P. Gawronski B. (2015). Gender differences in responses to moral dilemmas: a process dissociation analysis. Personal. Soc. Psychol. Bull. 41, 696–713. doi: 10.1177/0146167215575731, PMID: 25840987

[ref19] Fumagalli M. Ferrucci R. Mameli F. Marceglia S. Mrakic-Sposta S. Zago S.. (2010). Gender-related differences in moral judgments. Cogn. Process. 11, 219–226. doi: 10.1007/s10339-009-0335-2, PMID: 19727878

[ref20] Graham J. Nosek B. A. Haidt J. Iyer R. Koleva S. Ditto P. H. (2011). Mapping the moral domain. J. Pers. Soc. Psychol. 101, 366–385. doi: 10.1037/a0021847, PMID: 21244182 PMC3116962

[ref21] Greene J. D. Nystrom L. E. Engell A. D. Darley J. M. Cohen J. D. (2004). The neural bases of cognitive conflict and control in moral judgment. Neuron 44, 389–400. doi: 10.1016/j.neuron.2004.09.027, PMID: 15473975

[ref22] Greene J. D. Sommerville R. B. Nystrom L. E. Darley J. M. Cohen J. D. (2001). An fMRI investigation of emotional engagement in moral judgment. Science 293, 2105–2108. doi: 10.1126/science.1062872, PMID: 11557895

[ref23] Haidt J. (2007). The new synthesis in moral psychology. Science 316, 998–1002. doi: 10.1126/science.1137651 17510357

[ref24] Hoffman M. L. (1975). Sex differences in moral internalization and values. J. Pers. Soc. Psychol. 32, 720–729. doi: 10.1037/0022-3514.32.4.720, PMID: 1185510

[ref25] Hügelschäfer S. Achtziger A. (2014). On confident men and rational women: It’s all on your mind (set). J. Econ. Psychol. 41, 31–44. doi: 10.1016/j.joep.2013.04.001

[ref26] Humphries M. L. Parker B. L. Jagers R. J. (2000). Predictors of moral reasoning among African American children: a preliminary study. J. Black Psychol. 26, 51–64. doi: 10.1177/0095798400026001003

[ref27] Koenigs M. Young L. Adolphs R. Tranel D. Cushman F. Hauser M.. (2007). Damage to the prefrontal cortex increases utilitarian moral judgements. Nature 446, 908–911. doi: 10.1038/nature05631, PMID: 17377536 PMC2244801

[ref28] Lanteri A. Chelini C. Rizzello S. (2008). An experimental investigation of emotions and reasoning in the trolley problem. J. Bus. Ethics 83, 789–804. doi: 10.1007/s10551-008-9665-8

[ref29] Leary M. R. (2019). Self-presentation: Impression management and interpersonal behavior. New York: Routledge.

[ref30] Leary M. R. Kowalski R. M. (1990). Impression management: a literature review and two-component model. Psychol. Bull. 107, 34–47. doi: 10.1037/0033-2909.107.1.34

[ref31] Lee M. Sul S. Kim H. (2018). Social observation increases deontological judgments in moral dilemmas. Evol. Hum. Behav. 39, 611–621. doi: 10.1016/j.evolhumbehav.2018.06.004

[ref32] Lucas B. J. Livingston R. W. (2014). Feeling socially connected increases utilitarian choices in moral dilemmas. J. Exp. Soc. Psychol. 53, 1–4. doi: 10.1016/j.jesp.2014.01.011

[ref33] McHugh C. McGann M. Igou E. R. Kinsella E. L. (2022). Moral judgment as categorization (MJAC). Perspect. Psychol. Sci. 17, 131–152. doi: 10.1177/1745691621990636, PMID: 34264152 PMC8785282

[ref34] McNamara R. A. Willard A. K. Norenzayan A. Henrich J. (2019). Weighing outcome vs. intent across societies: how cultural models of mind shape moral reasoning. Cognition 182, 95–108. doi: 10.1016/j.cognition.2018.09.008, PMID: 30227333

[ref35] Moore A. B. Clark B. A. Kane M. J. (2008). Who shalt not kill? Individual differences in working memory capacity, executive control, and moral judgment. Psychol. Sci. 19, 549–557. doi: 10.1111/j.1467-9280.2008.02122.x, PMID: 18578844

[ref36] Rom S. C. Conway P. (2018). The strategic moral self: self-presentation shapes moral dilemma judgments. J. Exp. Soc. Psychol. 74, 24–37. doi: 10.1016/j.jesp.2017.08.003

[ref37] Rudman L. A. Glick P. (1999). Feminized management and backlash toward agentic women: the hidden costs to women of a kinder, gentler image of middle managers. J. Pers. Soc. Psychol. 77, 1004–1010. doi: 10.1037/0022-3514.77.5.1004, PMID: 10573877

[ref38] Schein C. (2020). The importance of context in moral judgments. Perspect. Psychol. Sci. 15, 207–215. doi: 10.1177/1745691620904083 32129715

[ref39] Sylvia T. Guy K. Sarah M. Julian S. Neil L. Miles H.. (2013). Beta adrenergic blockade reduces utilitarian judgement. Biol. Psychol. 92, 323–328. doi: 10.1016/j.biopsycho.2012.09.005, PMID: 23085134 PMC3573226

[ref40] Waldmann M. R. Nagel J. Wiegmann A. (2012). “Moral judgment” in The oxford handbook of thinking and reasoning Eds. KJ Holyoak, RG Morrison (New York: Oxford University Press), 364–389.

[ref41] Walker L. J. (1984). Sex differences in the development of moral reasoning: a critical review. Child Dev. 55, 677–691. doi: 10.2307/1130121

